# Neurosift: DANDI exploration and NWB visualization in the browser

**DOI:** 10.21105/joss.06590

**Published:** 2024-05-27

**Authors:** Jeremy Magland, Jeff Soules, Cody Baker, Benjamin Dichter

**Affiliations:** 1 Center for Computational Mathematics, Flatiron Institute, United States of America; 2 CatalystNeuro, United States of America

## Summary

Neurosift, a browser-based visualization tool, is designed for the interactive exploration of Neurodata Without Borders (NWB) files, whether stored locally, on remote servers, or within the Distributed Archives for Neurophysiology Data Integration (DANDI). NWB (Rübel et al., 2022; Teeters et al., 2015) is an open data standard for neurophysiology that enables the sharing, archiving, and analysis of various types of neurophysiology data. DANDI (Rübel et al., 2022) is a cloud-based platform that supports the storage, sharing, and analysis of neurophysiology data including NWB files. With Neurosift integration, users browsing DANDI can easily open any NWB file in the browser and explore its contents, including timeseries data, images, and more. Neurosift can also be used to browse the DANDI database or individual Dandisets. Overall, Neurosift simplifies the visualization and exploration of complex NWB file structures, making it a valuable tool for neuroscientists.

## Statement of need

In the evolving field of neuroscience research, the ability to manage and share complex data sets is crucial. NWB has emerged as a standard for neurophysiology data, aimed at facilitating data sharing, storage, and analysis. However, the specialized nature of the NWB format necessitates tools that can provide intuitive interfaces for researchers to explore their data effectively. Neurosift is designed to address this need.

Because files found on DANDI can often be large and unwieldy, various tools have emerged to address this issue by streaming portions of the NWB file without the need to download the entire file. One such tool is NWB Widgets (Dichter, 2022), which provides a suite of interactive widgets for visualizing NWB data within Jupyter notebooks, enabling users to navigate the hierarchical structure of NWB files and directly visualize specific data elements. This package was a large part of the inspiration for Neurosift. The main difference is that NWB Widgets is a Python package that runs within interactive Python environments, while Neurosift is a browser-based tool that can be used without any installation. These two tools cater to different use cases, with Neurosift being more accessible to a wider audience, and being better suited for integration with DANDI.

## Functionality

**Figure 1:**
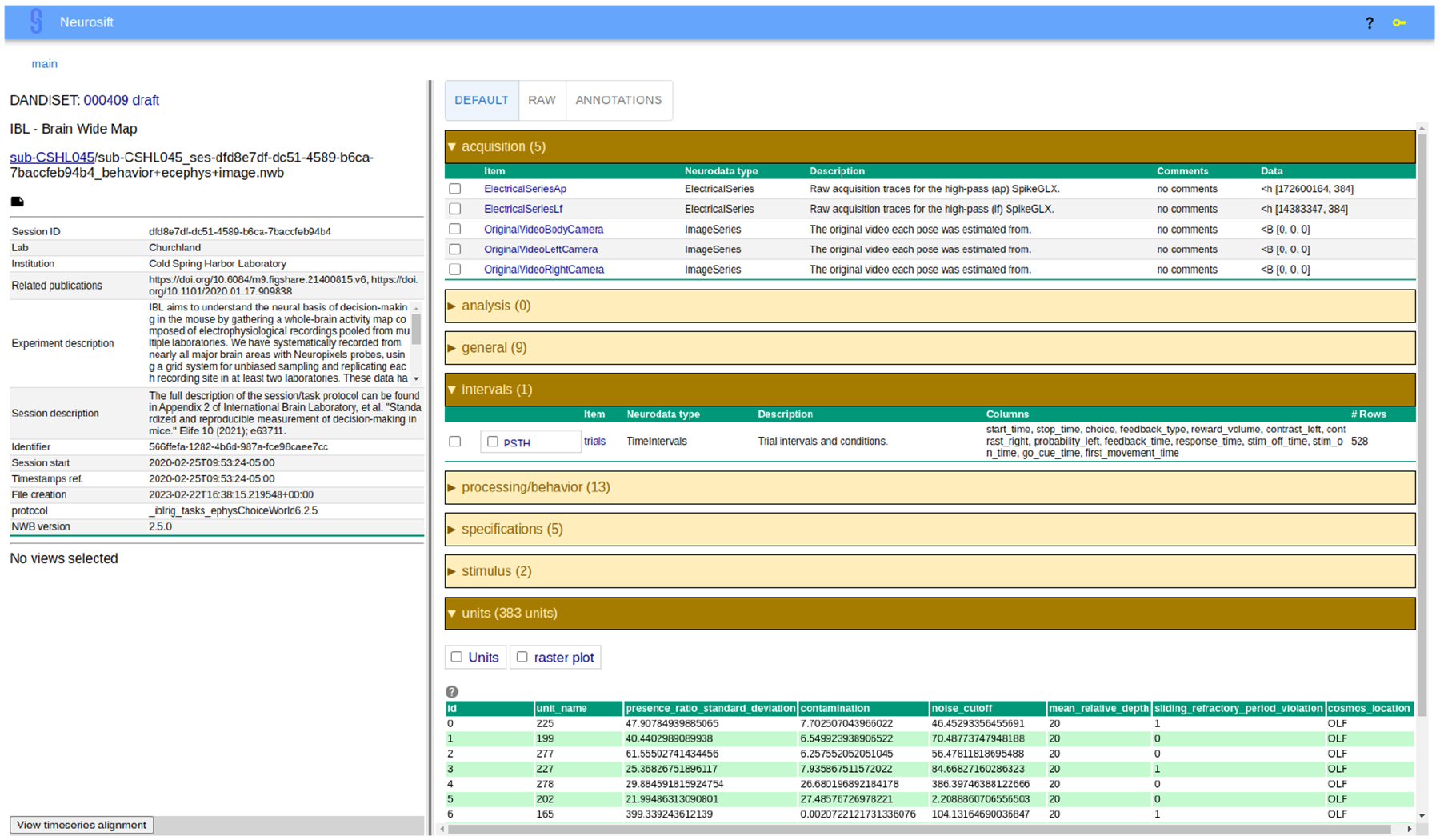
Screenshot of Neurosift displaying a NWB file from the ‘IBL - Brain Wide Map’ Dandiset (ID 000409), showcasing the tool’s capability to navigate and visualize the hierarchical structure of neurophysiological data. Visible are expanded panels for *ElectricalSeries* and *ImageSeries* objects, alongside a *TimeIntervals* object and a *Units* table.

**Figure 2:**
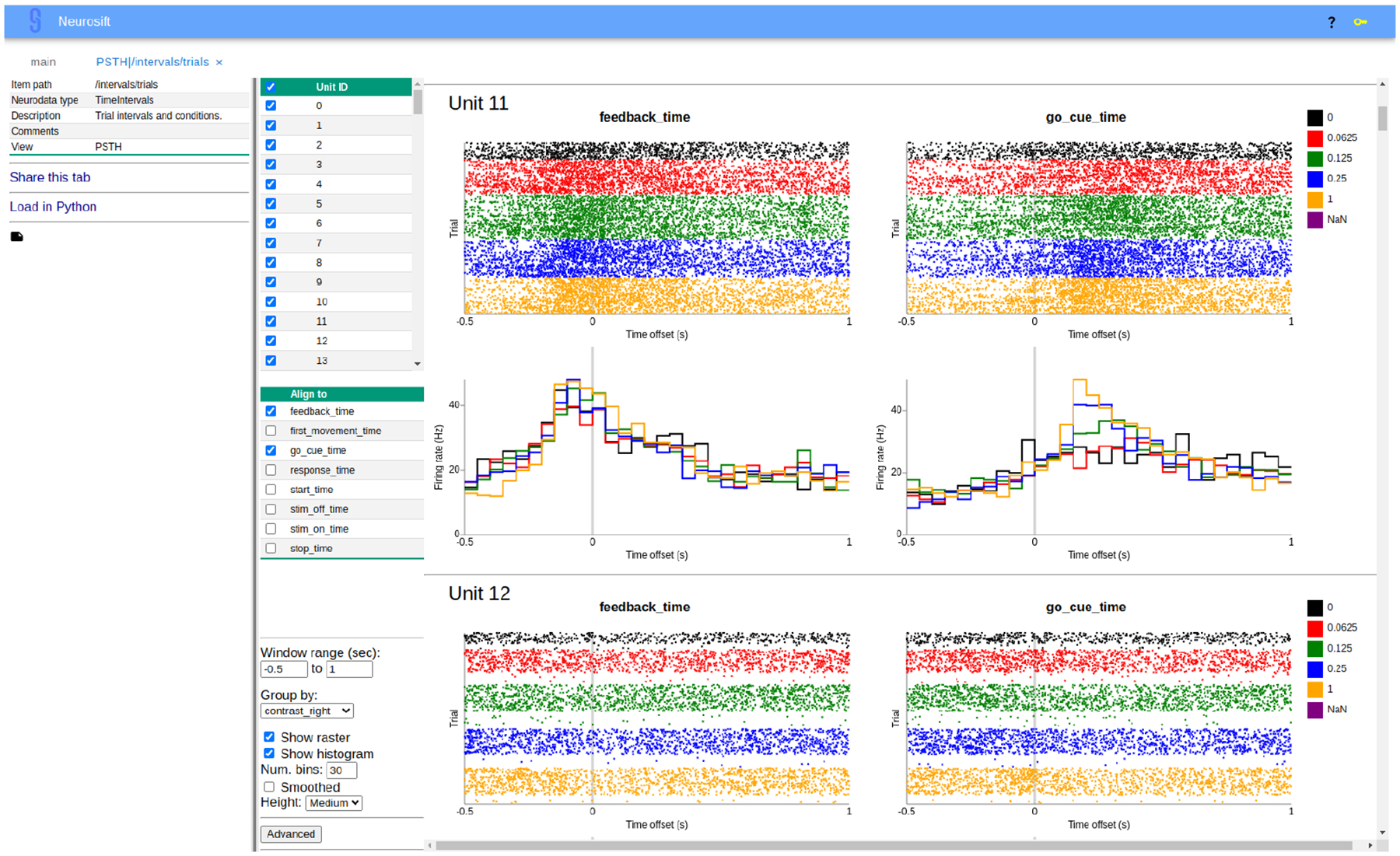
Interactive Peri-stimulus Time Histogram (PSTH) visualization in Neurosift, enabling users to select neural units, time alignment variables, and customize options like window range, histogram bin count, and grouping variables.

**Figure 3:**
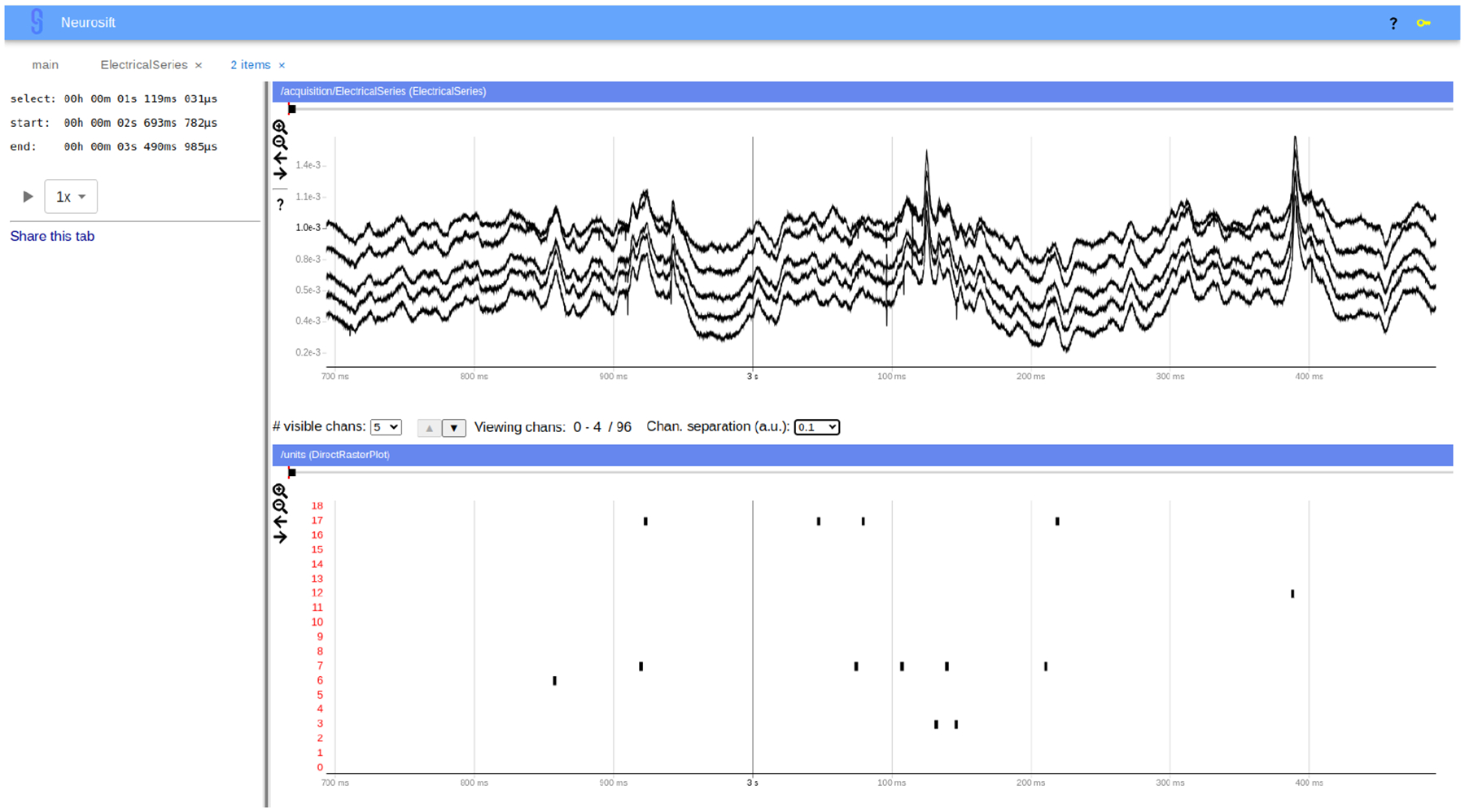
Synchronized view within Neurosift demonstrating the interactive alignment between an *ElectricalSeries* and a *Spike Raster Plot*. This feature allows users to seamlessly zoom and pan across both visualizations, maintaining a coherent temporal perspective across different data types.

Neurodata Without Borders files are structured hierarchically and encapsulate various “neurodata” types that reflect different aspects of neurophysiological experiments. These types range from *BehavioralEvents*, which record discrete actions or occurrences within experiments, to data structures like *Fluorescence*, *ImageSegmentation*, and *RoiResponseSeries*, which are key data types in optical neurophysiology. Other neurodata types include *ElectricalSeries* for electrophysiological signals and *Units* for spike times of neurons. Neurosift allows interactive navigation of this hierarchical structure (Figure 1) and provides plugin visualizations for many of these types (Figure 2). It also facilitates the creation of composite views by allowing users to select and synchronize multiple data types within the same interface (Figure 3). This synchronization extends to navigation actions such as zooming and panning, where different sub-windows, each displaying a different aspect of the data, maintain a shared time axis. These views can then be shared with others as a URL.

## Architecture and technical innovation

Neurosift is a *static* React/TypeScript website, meaning that it is delivered to the user’s browser exactly as stored, without the need for dynamic server-side processing of requests. This approach simplifies deployment and maintenance; it can be deployed to any static hosting service.

The main technical challenge in developing Neurosift was the requirement to lazy-load data objects from remote NWB files that are built on the complex HDF5 format. While HDF5’s efficient data organization is ideal for the large, multidimensional datasets typical in neurophysiology, its primary implementations are in the C language. This necessitates a creative solution to enable efficient web-based access to these files. To bridge this gap, Neurosift leverages WebAssembly to run compiled C code in the browser, specifically utilizing a modified version of the h5wasm (Maranville, 2022) library. Unlike the unmodified h5wasm, which primarily handles fully downloaded files, Neurosift’s fork introduces an innovative approach to efficiently read data chunks from remote files. This allows for partial data reads without the need for a prior download of the entire file. This solution showcases the potential of WebAssembly in overcoming challenges associated with web-based data analysis tools.

## Future directions

Looking forward, there is potential to expand Neurosift’s capabilities with enhanced visualizations and support for additional data types. Additionally, we are adding support for Zarr (Miles, 2024), a cloud-friendly alternative to HDF5 as a storage backend for NWB files.

## Conclusion

Neurosift makes neurophysiology data more accessible to scientists. By facilitating the exploration of complex datasets directly within a browser, it lowers the barrier to entry for data analysis and fosters collaborative research efforts.

## Data Availability

Review Repository Archive
